# Association between the sarcopenia index and abnormal liver function in the adult population in the United States: a cross-sectional study

**DOI:** 10.3389/fmed.2023.1266253

**Published:** 2023-11-21

**Authors:** Jian Xu, Zhi-Xiang Xu, Qi-Fan Yang, Jing Zhuang, Xin Zhu, Jun Yao

**Affiliations:** Department of Gastroenterology, The Affiliated People’s Hospital of Jiangsu University, Zhenjiang, Jiangsu, China

**Keywords:** sarcopenia index, abnormal liver function, adult population, cross-sectional study, NHANES

## Abstract

**Background:**

The objective of this study was to explore the association between the sarcopenia index and abnormal liver function in adult individuals in the United States.

**Methodology:**

This study employed a rigorous cross-sectional analysis of data derived from the National Health and Nutrition Examination Survey (NHANES) conducted between 2017 and 2018. The primary objective was to investigate the correlation between the sarcopenia index and abnormal liver function. To achieve this, an advanced multivariate regression model was utilized, allowing for comprehensive analysis and meticulous adjustment of relevant variables. To ensure the robustness of the findings, a visually appealing smooth curve was constructed, and a two-stage regression model was applied for validation. Additionally, a detailed gender-stratified analysis was conducted to further explore the association between the sarcopenia index and abnormal liver function within distinct subgroups.

**Results:**

Through our rigorous participant selection process, a total of 1756 individuals were included in the study. The meticulously adjusted multivariate regression model revealed a significant negative association between the sarcopenia index and abnormal liver function, with an adjusted odds ratio (OR) of 0.73 and a 95% confidence interval (CI) ranging from 0.63 to 0.86. The robustness of this association was further supported by the visually appealing smooth curve plot. Moreover, in the gender-stratified subgroup analysis, after meticulous adjustment for confounding factors, notable differences in this association emerged (males: OR = 0.8, 95% CI: 0.66–0.98; females: OR = 0.61, 95% CI: 0.47–0.79).

**Conclusion:**

This cross-sectional study yields robust evidence indicating a negative correlation between the sarcopenia index and abnormal liver function, predominantly observed among females.

## 1 Introduction

Sarcopenia is a condition characterized by the progressive reduction in muscle mass and strength, affecting both older adults and the general adult population (1, 2). Recent evidence suggests that sarcopenia can have significant effects on various organs and systems, including liver (3, 4).

The liver, as one of the primary organs responsible for metabolism in the human body, plays a vital role in various important functions, including processing substances, detoxification, breaking down fatty acids, and producing bile (5, 6). When the liver is unable to perform its normal physiological functions, it is referred to as abnormal liver function. Common pathological changes associated with abnormal liver function include fatty liver, cirrhosis, and liver cancer, among others (7, 8). The most common symptoms include elevated liver enzymes, and changes in the structure of the liver (9).

The association between sarcopenia and abnormal liver function is not fully understood, but several potential mechanisms have been identified. Metabolic and endocrine changes, such as insulin resistance, disrupted fatty acid metabolism, and inflammation, may adversely affect liver function in muscle atrophy patients (10). Additionally, hormones, cytokines, and neural regulation pathways influenced by sarcopenia may impact liver physiology (11).

Sarcopenia index is a quantitative measure used to assess the degree of muscle loss and functional decline in individuals (12). However, the nature and extent of its relationship with abnormal liver function have not been conclusively established in the existing literature. In this study, we conducted a rigorous analysis utilizing the data from the 2017–2018 cycle of the NHANES to investigate the association between the sarcopenia index and abnormal liver function in adults. This research aims to provide a theoretical foundation and guidance for further understanding the impact of sarcopenia on liver health, ultimately leading to improved healthcare recommendations for individuals.

## 2 Methods

### 2.1 Study population

**Figure 1:**
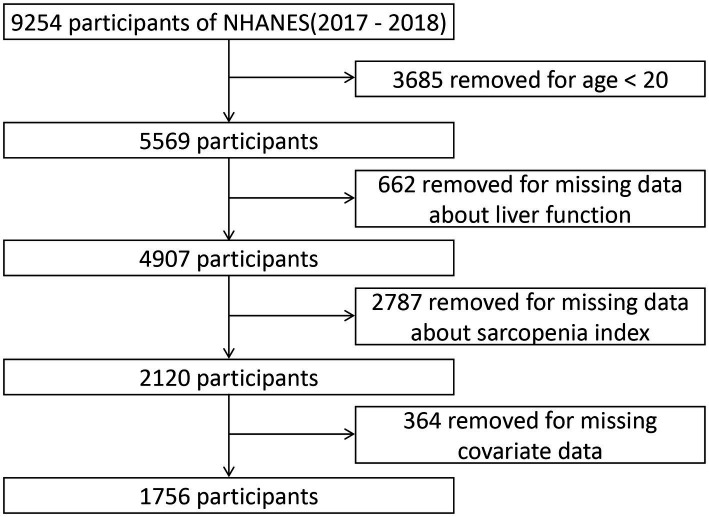
Flowchart illustrating the criteria for participant selection and exclusion in our study cohort.

In the NHANES, a biennial national health survey conducted by the National Center for Health Statistics (NCHS), comprehensive data on the health status of the population in the United States is collected (13). This publicly accessible dataset provides extensive information on nutrition and overall health of the general population. The survey data has been ethically approved by the NCHS Institutional Review Board, allowing researchers and users to access it. For this study, we utilized the aggregated NHANES data from the combined years of 2017 and 2018. Participants were excluded from the analysis if they met any of the following criteria: (1) age below 20 years, (2) absence of liver function indicators, (3) inability to calculate the sarcopenia index, and (4) missing covariate data. The final analysis included a total of 1756 individuals, as depicted in Figure 1.

### 2.2 Sarcopenia index

The primary variable under investigation in this study is the sarcopenia index. NHANES utilizes dual-energy X-ray absorptiometry (DXA) to assess the appendicular lean mass (ALM), which represents the cumulative skeletal muscle mass of the limbs. The sarcopenia index is computed by dividing the ALM (measured in kilograms) by the BMI (body mass index), which is determined by dividing the weight (measured in kilograms) obtained during the physical examination by the square of the height (measured in meters) (10, 14).

### 2.3 Liver function measurement results

Blood samples from NHANES participants were meticulously collected at a mobile examination center. These invaluable samples were carefully preserved in small vials under optimal refrigeration conditions (2–8°C) and expeditiously transported to the esteemed Advanced Research and Diagnostic Laboratory (ARDL) at the University of Minnesota. Utilizing the cutting-edge Roche Cobas 6000 chemistry analyzer (Cobas 6000), the laboratory conducted a comprehensive analysis of serum liver function indicators (accessible on the official NHANES).^1^

The liver, a vital organ, is abundant in two crucial enzymes: alanine aminotransferase (ALT) and aspartate aminotransferase (AST). Elevated serum levels of these enzymes serve as markers of liver cell damage (15). Additionally, alkaline phosphatase (ALP) and gamma-glutamyl transferase (GGT) are also markers of abnormal liver function (16). Furthermore, the liver plays a pivotal role in the metabolism of bilirubin, encompassing its uptake, conjugation, and excretion. Dysfunctions in any of these processes can contribute to elevated levels of total bilirubin (TBIL) (17). In this study, abnormal liver function is defined as elevated levels of ALT, AST, ALP, GGT, or TBIL that exceed the upper limits of the established normal range (AST: 0–37 U/L for males, 0–31 U/L for females; ALT: 0–40 U/L for males, 0–31 U/L for females; ALP: 40–129 U/L for males, 35–104 U/L for females; GGT: 11–51 U/L for males, 7–33 U/L for females; TBIL: 1.0 mg/dL).

### 2.4 Potential covariates

Based on previous studies, the following variables were included as covariates in our analysis: age (<40 years/≥40 years), gender (male/female), race (Hispanic White/Non-Hispanic Black/Mexican American/Other Hispanic/Other), smoking status (ever smoked at least 100 cigarettes/never smoked) (18), alcohol consumption (ever consumed any type of alcoholic beverage/never consumed) (19), hypertension (systolic blood pressure ≥ 140 mmHg/diastolic blood pressure ≥ 90 mmHg/clinically diagnosed hypertension/current use of antihypertensive medication), diabetes (clinically diagnosed diabetes/glycated hemoglobin ≥6.5%/current use of antidiabetic medication), cancer (clinically diagnosed cancer), gallstones (diagnosed with gallstones by a healthcare professional), physical activity level (being classified as active requires participating in a minimum of 75 min of vigorous-intensity exercise per week or 150 min of moderate-intensity exercise per week; being considered insufficiently active means engaging in fewer than 75 min of vigorous-intensity exercise per week or less than 150 min of moderate-intensity exercise per week; being classified as inactive means not engaging in any form of physical activity) (20), depression (total score ≥ 10 on a nine-item depression screening tool) (21), and hepatitis status (hepatitis B/hepatitis C).

### 2.5 Statistical analysis

Sampling weights were applied in the NHANES to account for the complex study design. We utilized the recommended 2-year sample weights (WTMEC2YR) provided by the Centers for Disease Control and Prevention for the mobile examination centers. Baseline characteristics and univariate analysis of abnormal liver function were presented as mean ± standard deviation for continuous variables, while categorical variables were expressed as percentages with a 95% confidence interval. Weighted one-way ANOVA and t-tests were applied to continuous variables, while chi-square tests were utilized for categorical variables to assess disparities among different categories of the sarcopenia index and the risk factors for abnormal liver function.

Three logistic regression models were employed to investigate the independent association between the sarcopenia index and abnormal liver function, with adjustments made for potential confounding factors. Model 1 remained unadjusted, while Model 2 included adjustments for age, gender, and race. Model 3 accounted for additional adjustments, including smoking status, alcohol consumption, hypertension, diabetes, cancer, gallstones, physical activity, depression and hepatitis status. To comprehensively explore this relationship, the sarcopenia index was analyzed both as a continuous variable and stratified into three categories (<0.663, 0.663–0.884, >0.884) based on tertiles. Furthermore, a two-stage linear regression model was utilized to assess the potential threshold effect of the sarcopenia index on abnormal liver function, as indicated by the fitted smooth curve plot. A testing approach was employed to identify the inflection point where the relationship between the sarcopenia index and abnormal liver function exhibited significant changes, optimizing the model likelihood within a predefined interval. Additionally, a log-likelihood ratio test was conducted to compare the fit of a linear regression model (piecewise linear regression model) with that of a two-stage linear regression model.

Lastly, a gender-stratified subgroup analysis was conducted to examine the relationship between the sarcopenia index and abnormal liver function within different gender groups.

All statistical analyses were conducted using the R, Stata, and EmpowerStats software packages. Statistical significance was defined as a *p*-value of <0.05 (two-tailed).

## 3 Results

### 3.1 Participant characteristics

A total of 1,756 adult Americans participated in the study, with 53.09% being younger than 40 years old and 46.91% being 40 years old or older. Female participants made up 50.16% and male participants 49.84%. Table 1 presents participant characteristics at baseline, categorized by distinct sarcopenia index levels. Approximately 24.86% of the participants had abnormal liver function. The proportions of participants with abnormal liver function were 31.28, 24.43, and 20.36% in the groups with sarcopenia index <0.664, 0.664–0.884, and > 0.884, respectively.

**Table 1:** Characteristics of participants stratified by sarcopenia index tertiles (tertiles 1–3).

	Total	T1 (< 0.664)	T2 (0.664–0.884)	T3 (> 0.884)	*P*-value
Sample size (example)	1,756	585	585	586	
Sarcopenia index	0.812 ± 0.195	0.584 ± 0.055	0.773 ± 0.067	1.019 ± 0.103	< 0.001
Liver function					< 0.001
Normal	75.14 (72.16–77.89)	68.72 (62.98–73.94)	75.57 (70.55–79.98)	79.64 (74.63–83.88)	
Abnormal	24.86 (22.11–27.84)	31.28 (26.06–37.02)	24.43 (20.02–29.45)	20.36 (16.12–25.37)	
Age (years)					< 0.001
< 40	53.09 (49.65–56.49)	46.27 (40.7–51.93)	54.28 (48.4–60.04)	57.22 (51.12–63.12)	
≥ 40	46.91 (43.51–50.35)	53.73 (48.07–59.3)	45.72 (39.96–51.6)	42.78 (36.88–48.88)	
Gender					< 0.001
Male	49.84 (46.44–53.24)	0.34 (0.11–1.04)	41.32 (35.76–47.12)	95.1 (91.98–97.05)	
Female	50.16 (46.76–53.56)	99.66 (98.96–99.89)	58.68 (52.88–64.24)	4.9 (2.95–8.02)	
Race					< 0.001
Mexican American	11.61 (10.15–13.25)	15.13 (12.37–18.38)	12.85 (10.27–15.95)	7.83 (5.85–10.41)	
Other Hispanic	8.35 (7.05–9.86)	9.36 (7.1–12.25)	9.21 (6.94–12.12)	6.82 (4.95–9.32)	
Non-Hispanic White	58.46 (55.36–61.49)	52.48 (46.9–58.01)	57.8 (52.38–63.04)	63.58 (58.55–68.34)	
Non-Hispanic Black	10.3 (9.1–11.64)	9.89 (7.86–12.36)	8.09 (6.37–10.22)	12.58 (10.38–15.17)	
Other race—including multi-racial	11.28 (9.73–13.05)	13.14 (10.4–16.46)	12.05 (9.09–15.81)	9.19 (7.18–11.68)	
Smoke					< 0.001
No	60.26 (56.86–63.57)	68.13 (62.54–73.24)	60.06 (54.29–65.56)	54.44 (48.41–60.35)	
Yes	39.74 (36.43–43.14)	31.87 (26.76–37.46)	39.94 (34.44–45.71)	45.56 (39.65–51.59)	
Drink alcohol					0.005
No	7.78 (6.22–9.71)	11.05 (8.01–15.05)	6.35 (4.34–9.2)	6.57 (4.09–10.39)	
Yes	92.22 (90.29–93.78)	88.95 (84.95–91.99)	93.65 (90.8–95.66)	93.43 (89.61–95.91)	
Hypertension					0.606
No	79.23 (76.37–81.83)	78.36 (73.54–82.5)	80.59 (75.62–84.75)	78.7 (73.36–83.22)	
Yes	20.77 (18.17–23.63)	21.64 (17.5–26.46)	19.41 (15.25–24.38)	21.3 (16.78–26.64)	
Diabetes					< 0.001
No	92 (90.13–93.54)	86.77 (82.59–90.06)	93.52 (90.4–95.68)	94.63 (91.4–96.68)	
Yes	8 (6.46–9.87)	13.23 (9.94–17.41)	6.48 (4.32–9.6)	5.37 (3.32–8.6)	
Cancer					0.058
No	95.38 (93.42–96.78)	93.51 (88.84–96.3)	96.1 (93.37–97.73)	96.18 (92.09–98.2)	
Yes	4.62 (3.22–6.58)	6.49 (3.7–11.16)	3.9 (2.27–6.63)	3.82 (1.8–7.91)	
Gallstone					< 0.001
No	93.36 (91.7–94.71)	85.51 (81.25–88.92)	96.26 (94.25–97.59)	96.77 (93.84–98.33)	
Yes	6.64 (5.29–8.3)	14.49 (11.08–18.75)	3.74 (2.41–5.75)	3.23 (1.67–6.16)	
Physical activity					< 0.001
Never	43.47 (40.14–46.86)	54.43 (48.7–60.04)	42.6 (36.95–48.44)	35.89 (30.44–41.74)	
Less active	8.7 (6.95–10.84)	6.9 (4.68–10.08)	9.86 (6.97–13.78)	9.04 (6.01–13.4)	
Active	47.83 (44.44–51.24)	38.67 (33.26–44.37)	47.54 (41.82–53.33)	55.06 (49.08–60.9)	
Depression					0.015
No	92.01 (90.19–93.52)	89.13 (85.81–91.75)	93.7 (90.8–95.73)	92.72 (88.96–95.26)	
Yes	7.99 (6.48–9.81)	10.87 (8.25–14.19)	6.3 (4.27–9.2)	7.28 (4.74–11.04)	
Hepatitis status					0.334
No	97.55 (96.14–98.46)	98.08 (96.56–98.94)	97.88 (95.48–99.02)	96.86 (93.26–98.57)	
Yes	2.45 (1.54–3.86)	1.92 (1.06–3.44)	2.12 (0.98–4.52)	3.14 (1.43–6.74)	

T, tertile; SD, standard deviation. Means ± SD for continuous variables, and *p*-values were calculated using the weighted one-way ANOVA. Weighted proportion (95% confidence interval) for categorical variables, and *p*-values calculated using the weighted Chi-square test.

### 3.2 Univariate analysis

Among the 1,756 participants, 1,286 (73.23%) were categorized as having normal liver function, while 470 (26.77%) were categorized as having abnormal liver function. A statistically significant difference was not found between the two groups in regards to gender, race, alcohol consumption, cancer, gallstones, physical activity, or depression between the two groups (*p* > 0.05). However, the differences between the two groups were statistically significant in terms of sarcopenia index, age, smoking status, hypertension, diabetes, and hepatitis status (*p* < 0.05), as shown in Table 2.

**Table 2:** Univariate analysis between patients with and without abnormal liver function.

	Total	Normal liver function	Abnormal liver function	*P*-value
Sample size (example)	1,756	1,286	470	
Sarcopenia index	0.812 ± 0.195	0.823 ± 0.192	0.778 ± 0.198	< 0.001
Age (years)				< 0.001
< 40	53.09 (49.65–56.49)	55.59 (51.55–59.56)	45.5 (39.15–52.01)	
≥ 40	46.91 (43.51–50.35)	44.41 (40.44–48.45)	54.5 (47.99–60.85)	
Gender				0.900
Male	49.84 (46.44–53.24)	49.93 (45.96–53.89)	49.58 (43.05–56.12)	
Female	50.16 (46.76–53.56)	50.07 (46.11–54.04)	50.42 (43.88–56.95)	
Race				0.503
Mexican American	11.61 (10.15–13.25)	11.19 (9.54–13.09)	12.87 (10.02–16.38)	
Other Hispanic	8.35 (7.05–9.86)	8.37 (6.88–10.15)	8.29 (5.95–11.44)	
Non-Hispanic White	58.46 (55.36–61.49)	59.49 (55.91–62.96)	55.34 (49.11–61.41)	
Non-Hispanic Black	10.3 (9.1–11.64)	9.76 (8.43–11.27)	11.95 (9.46–15)	
Other race—including multi-racial	11.28 (9.73–13.05)	11.2 (9.42–13.26)	11.55 (8.65–15.26)	
Smoke				0.011
No	60.26 (56.86–63.57)	61.97 (58.01–65.78)	55.09 (48.45–61.56)	
Yes	39.74 (36.43–43.14)	38.03 (34.22–41.99)	44.91 (38.44–51.55)	
Drink alcohol				0.073
No	7.78 (6.22–9.71)	8.44 (6.52–10.87)	5.79 (3.79–8.76)	
Yes	92.22 (90.29–93.78)	91.56 (89.13–93.48)	94.21 (91.24–96.21)	
Hypertension				< 0.001
No	79.23 (76.37–81.83)	81.93 (78.74–84.73)	71.09 (64.69–76.75)	
Yes	20.77 (18.17–23.63)	18.07 (15.27–21.26)	28.91 (23.25–35.31)	
Diabetes				< 0.001
No	92 (90.13–93.54)	94.24 (92.13–95.81)	85.22 (80.71–88.83)	
Yes	8 (6.46–9.87)	5.76 (4.19–7.87)	14.78 (11.17–19.29)	
Cancer				0.157
No	95.38 (93.42–96.78)	94.98 (92.4–96.71)	96.62 (94.23–98.04)	
Yes	4.62 (3.22–6.58)	5.02 (3.29–7.6)	3.38 (1.96–5.77)	
Gallstone				0.547
No	93.36 (91.7–94.71)	93.57 (91.59–95.1)	92.74 (89.23–95.17)	
Yes	6.64 (5.29–8.3)	6.43 (4.9–8.41)	7.26 (4.83–10.77)	
Physical activity				0.092
Never	43.47 (40.14–46.86)	43.88 (39.99–47.85)	42.23 (35.92–48.81)	
Less active	8.7 (6.95–10.84)	9.42 (7.27–12.12)	6.53 (4.27–9.85)	
Active	47.83 (44.44–51.24)	46.7 (42.76–50.68)	51.24 (44.7–57.75)	
Depression				0.610
No	92.01 (90.19–93.52)	92.2 (89.99–93.96)	91.44 (87.94–93.99)	
Yes	7.99 (6.48–9.81)	7.8 (6.04–10.01)	8.56 (6.01–12.06)	
Hepatitis status				< 0.001
No	97.55 (96.14–98.46)	98.79 (98–99.27)	93.82 (88.4–96.8)	
Yes	2.45 (1.54–3.86)	1.21 (0.73–2)	6.18 (3.2–11.6)	

SD, standard deviation. Means ± SD for continuous variables, and *p*-values were calculated using the weighted *t-*test. Weighted proportion (95% confidence interval) for categorical variables, and *p*-values calculated using the weighted Chi-square test.

### 3.3 Multiple regression analysis

Three multivariable logistic regression models were employed to investigate the association between the sarcopenia index and abnormal liver function (Table 3). Model 1 was unadjusted, while Model 2 controlled for age, sex, and race. Model 3 further adjusted for additional factors, including smoking status, alcohol consumption, hypertension, diabetes, cancer, gallstones, physical activity, depression and hepatitis status. Consistently across all three models, a significant inverse relationship was observed between the sarcopenia index and abnormal liver function. For each 0.1-unit increase in the sarcopenia index, the impact on abnormal liver function was as follows: Model 1 (OR = 0.88, 95% CI: 0.81–0.97), Model 2 (OR = 0.72, 95% CI: 0.62–0.85), and Model 3 (OR = 0.73, 95% CI: 0.63–0.86).

**Table 3:** Association between the sarcopenia index and the odds of abnormal liver function.

	Model 1 OR (95% CI)	Model 2 OR (95% CI)	Model 3 OR (95% CI)
Sarcopenia index (per 0.1 U)	0.88 (0.81–0.97)	0.72 (0.62–0.85)	0.73 (0.63–0.86)
Sarcopenia index (Tertiles 1–3)			
T1 (< 0.664)	Reference	Reference	Reference
T2 (0.664–0.884)	0.71 (0.49–1.02)	0.48 (0.30–0.77)	0.52 (0.32–0.84)
T3 (> 0.884)	0.56 (0.38–0.82)	0.24 (0.13–0.46)	0.28 (0.15–0.55)
P for trend	< 0.01	< 0.01	< 0.01

Model 1: adjusted for no covariates. Model 2: adjusted for age, gender, and race. Model 3: adjusted for age, gender, race, smoking status, alcohol consumption, hypertension, diabetes, cancer, gallstones, physical activity, depression, and hepatitis. U, unit; OR, odds ratio; CI, confidence interval; T, tertile.

When assessing various levels of the sarcopenia index, individuals in the second tertile exhibited no significant difference in the probability of abnormal liver function compared to those with the sarcopenia index levels in the lowest tertile, as indicated by the findings in Model 1 (OR = 0.71, 95% CI: 0.49–1.02). However, in Model 2 (OR = 0.48, 95% CI: 0.30–0.77) and Model 3 (OR = 0.52, 95% CI: 0.32–0.84), they demonstrated a reduced likelihood. Notably, individuals in the highest tertile had a significantly lower likelihood of abnormal liver function in Model 1 (OR = 0.56, 95% CI: 0.38–0.82), Model 2 (OR = 0.24, 95% CI: 0.13–0.46), and Model 3 (OR = 0.28, 95% CI: 0.15–0.55). Furthermore, the trend *p*-values for all three models were less than 0.01.

### 3.4 Nonlinear analysis

**Figure 2:**
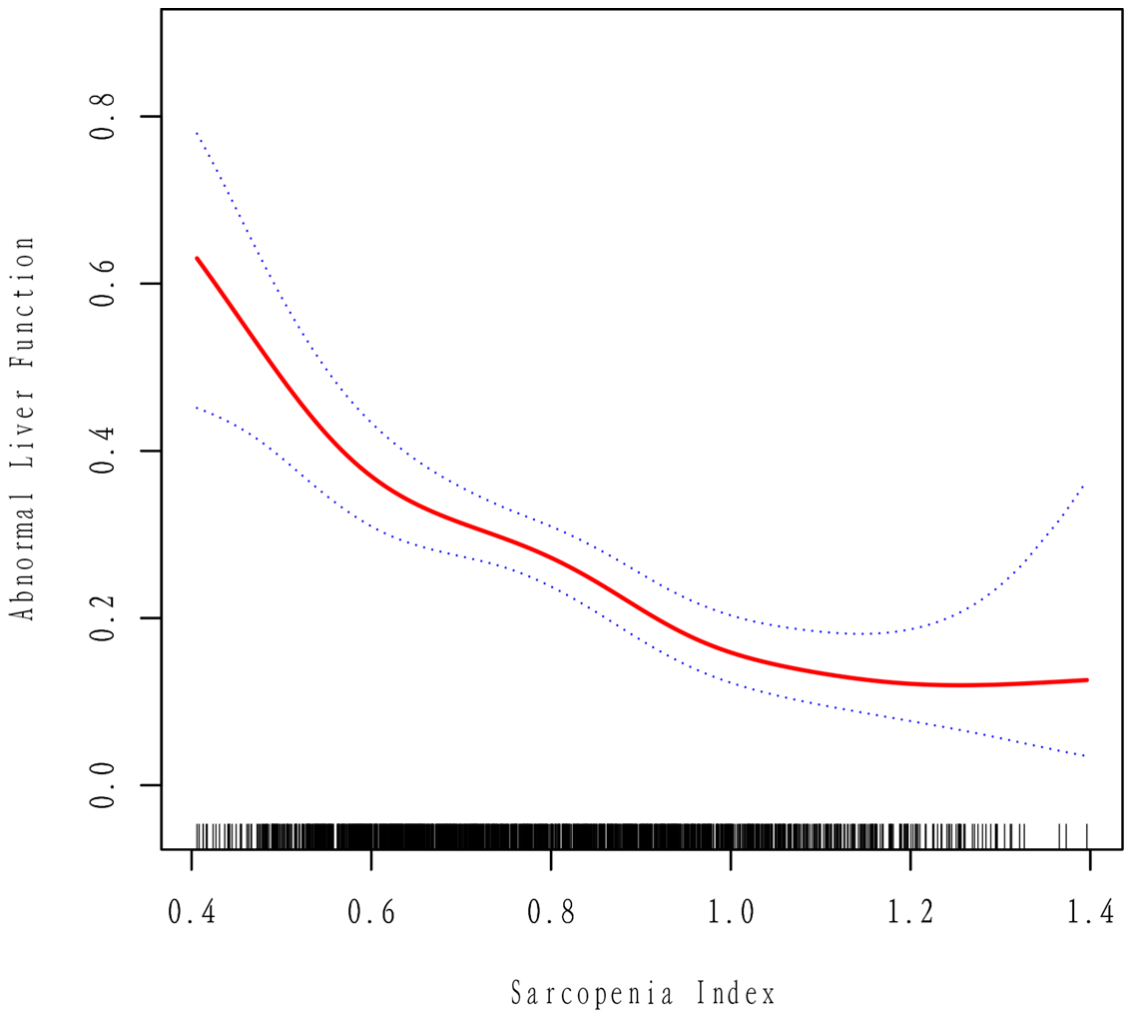
Correlation between the sarcopenia index and abnormal liver function, after adjusting for all covariates, as depicted by a smoothed curve. The two dashed lines represent the upper and lower limits of the 95% confidence interval.

Figure 2 illustrates a notable nonlinear relationship between the sarcopenia index and abnormal liver function, after adjusting for all relevant covariates. The probability of developing abnormal liver function gradually decreases as the sarcopenia index increases. Subsequently, a two-stage linear regression model was constructed by identifying the optimal inflection point that maximized the model likelihood. A likelihood ratio test was conducted to compare the two-stage linear regression model with a linear regression model, and the results indicated no statistically significant difference (*p* > 0.05). These findings suggest that the presence of a significant inflection point is not supported (Table 4).

**Table 4:** Threshold analysis of the sarcopenia index on abnormal liver function.

	Adjust OR (95% CI)	*P*
Model 1: a linear regression model (per 0.1 U)	0.73 (0.63–0.86)	<0.01
Model 2: two-piece linear regression model		
Inflection point	0.624	
Sarcopenia index <0.614 (per 0.1 U)	0.47 (0.25–0.90)	0.02
sarcopenia index >0.614 (per 0.1 U)	0.81 (0.68–0.96)	0.02
Log likelihood ratio test	> 0.05	

All models were adjusted for age, sex, race, smoking status, alcohol consumption, hypertension, diabetes, cancer, gallstones, physical activity, depression and hepatitis. U, unit; OR, odds ratio; CI, confidence interval.

### 3.5 Subgroup analysis

When stratified by gender, in multivariable-adjusted models among male participants, we identified a statistically significant difference (*p* < 0.05) in the association between the sarcopenia index and abnormal liver function. Compared to individuals in the lowest tertile of the sarcopenia index, those in the highest tertile exhibited a reduced likelihood of experiencing abnormal liver function with an odds ratio of 0.47 (95% CI, 0.25–0.87). However, among female participants, for every 0.1 unit increase in the muscle depletion index, the probabilities of having abnormal liver function were 0.61 (95% CI, 0.49–0.76), 0.61 (95% CI, 0.48–0.78), and 0.61 (95% CI, 0.47–0.79) in Model1, Model2, and Model3, respectively. The negative correlation between the sarcopenia index and abnormal liver function persisted. In all three adjusted models, compared to participants at the lowest tertile of the sarcopenia index, those at the second and third tertiles had a reduced likelihood of experiencing abnormal liver function. The trend *p*-values for all three models were less than 0.01 (Table 5).

**Table 5:** Associations between sarcopenia index and odds of abnormal liver function by gender.

		Model 1 OR (95% CI)	Model 2 OR (95% CI)	Model 3 OR (95% CI)
Stratified by gender				
Male	Sarcopenia index (per 0.1 U)	0.81 (0.67–0.98)	0.81 (0.66–1)	0.8 (0.66–0.98)
	Sarcopenia index (Tertiles 1–3)			
	T1 (< 0.884)	Reference	Reference	Reference
	T2 (0.884–0.998)	0.51 (0.30–0.87)	0.51 (0.30–0.88)	0.60 (0.35–1.06)
	T3 (> 0.998)	0.48 (0.27–0.84)	0.47 (0.25–0.90)	0.47 (0.25–0.87)
	P for trend	< 0.01	< 0.01	< 0.01
Female	Sarcopenia index (per 0.1 U)	0.61 (0.49–0.76)	0.61 (0.48–0.78)	0.61 (0.47–0.79)
	Sarcopenia index (Tertiles 1–3)			
	T1 (< 0.593)	Reference	Reference	Reference
	T2 (0.593–0.674)	0.54 (0.33–0.89)	0.55 (0.32–0.93)	0.57 (0.33–0.97)
	T3 (> 0.674)	0.35 (0.21–0.61)	0.37 (0.21–0.67)	0.38 (0.21–0.70)
	P for trend	< 0.01	< 0.01	< 0.01

Model 1: adjusted for no covariates. Model 2: adjusted for age, and race. Model 3: adjusted for age, race, smoking status, alcohol consumption, hypertension, diabetes, cancer, gallstones, physical activity, depression and hepatitis. U, unit; OR, odds ratio; CI, confidence interval; T, tertile.

## 4 Discussion

This study aimed to explore the relationship between the sarcopenia index and abnormal liver function. The results demonstrated a significant inverse correlation between the sarcopenia index and the occurrence of abnormal liver function, with a notable decrease in the incidence as the sarcopenia index increased. This association was further substantiated by the fitting of a smooth curve, providing additional evidence. Furthermore, gender-stratified analysis revealed a statistically significant relationship predominantly among females.

Sarcopenia, a condition characterized by muscle wasting, can exert substantial deleterious effects on multiple physiological organs, including the liver. Previous research endeavors have unveiled a compelling nexus between sarcopenia and the initiation as well as progression of hepatic pathologies, encompassing non-alcoholic fatty liver disease, cirrhosis, and hepatocellular carcinoma. In order to elucidate this correlation, a comprehensive inquiry encompassing 309 instances, led by Koo et al. (22), has definitively established a substantial relationship between diminished skeletal muscle mass and the histopathological gravity of non-alcoholic fatty liver disease. Additionally, sarcopenia has exhibited a pronounced correlation with non-alcoholic steatohepatitis within this context. Furthermore, a retrospective analysis comprising 8,361 subjects has discerned sarcopenia as a pivotal gauge of the severity of non-alcoholic fatty liver disease (23). Tandon et al. (24), in a systematic analysis, have explicated the proclivity of sarcopenia in influencing the prevalence and fatality rates associated with cirrhosis. In a meta-analysis incorporating findings from 22 studies, Tantai et al. (25) have underscored the escalating mortality rates in cirrhosis patients, concomitant with the severity and duration of sarcopenia. Notably, Perisetti et al. (26) have documented the early manifestation of sarcopenia in hepatocellular carcinoma, alongside its close correlation with mortality rates in hepatocellular carcinoma cases. These investigations corroborate our own research outcomes and provide further empirical substantiation, particularly in the quantification of the interrelation between sarcopenia index and abnormal liver function.

The potential mechanistic underpinnings delineating the nexus between sarcopenia and abnormal liver function encompass a spectrum of contributory facets, as follows: Firstly, sarcopenia may adversely affect liver function through insulin resistance. Sarcopenia results in decreased responsiveness of muscle tissue to insulin, leading to reduced glucose uptake and elevated blood sugar levels (10, 27). High insulin levels stimulate glucose production in the liver while inhibiting glycogen synthesis and utilization, causing excessive accumulation of glucose and fat in the liver, ultimately resulting in liver damage (28). Secondly, sarcopenia may adversely affect liver function through disruptions in fatty acid metabolism. Disruptions in fatty acid metabolism pose challenges to the liver, leading to oxidative stress and fat accumulation, triggering oxidative stress and inflammatory responses (29). This can result in liver cell injury and functional abnormalities. Thirdly, sarcopenia may affect liver function through inflammatory responses (29). The inflammatory responses triggered by sarcopenia may be associated with muscle tissue damage, release of inflammatory mediators, as well as inflammation in adipose tissue and oxidative stress caused by malnutrition. These inflammatory factors can enter the circulatory system and affect the liver, leading to liver cell damage and functional abnormalities. Fourthly, sarcopenia may adversely affect liver function through dysregulated hormone regulation. Sarcopenia contributes to insulin resistance, disrupting normal carbohydrate and fat metabolism, resulting in liver fat accumulation and abnormal cholesterol synthesis (30). Additionally, sarcopenia may lower testosterone and growth hormone levels, impacting fat metabolism and muscle tissue recovery, further negatively affecting liver function (31). Fifthly, sarcopenia may adversely affect liver function through dysregulated cytokine regulation. Sarcopenia leads to decreased muscle tissue and impaired function, which may trigger inflammatory responses and excessive release of cytokines. These inflammatory cytokines, such as TNF-α, IL-6, and C-reactive protein, can induce liver inflammation and fibrosis, causing liver cell damage and interfering with liver function (32). Furthermore, sarcopenia may also reduce anti-inflammatory factors, disrupting cytokine balance and exacerbating liver inflammation and functional abnormalities (32). These dysregulated mechanisms interact with each other, ultimately leading to the occurrence and development of abnormal liver function. Finally, sarcopenia may adversely affect liver function through abnormal neural regulation. The nervous system plays a crucial role in regulating metabolism, inflammatory responses, and hormonal balance in the body (33). Sarcopenia can lead to heightened sympathetic nervous system activity and diminished parasympathetic nervous system activity, disrupting the delicate balance of the neurophysiological milieu. This disruption may induce an upregulation of inflammatory and stress responses, thereby exerting an adverse influence on hepatic function (34). Additionally, sarcopenia may interfere with neural regulatory pathways between muscle and liver, affecting liver physiological regulation and metabolic activities. The occurrence of insulin resistance may also be one of the mechanisms by which sarcopenia affects liver function through neural regulation (34).

Subsequent investigations conducted among different genders have unveiled that the influence of the sarcopenia index on abnormal liver function is notably more pronounced within the female demographic. Several factors may contribute to the greater manifestation of abnormal liver function caused by sarcopenia in women. Firstly, females generally possess higher levels of body fat and lower muscle mass physiologically (35, 36). Under conditions where muscle mass changes are equivalent, the impact on liver function may be more evident. Additionally, the accumulation of body fat is closely associated with hepatic fat accumulation, which can lead to the development of fatty liver and non-alcoholic fatty liver disease (37). Consequently, this can trigger abnormal liver function. Secondly, this gender disparity could be attributed to the protective effects of estrogen on female liver function, which may enhance the association between the sarcopenia index and abnormal liver function in women (38, 39).

Moreover, amid the set of covariates ascertained in this inquiry, a statistically significant differentiation in terms of abnormal liver function coinciding with hypertension has been discerned. A working hypothesis posits that this phenomenon can be ascribed to heightened blood pressure inciting pro-inflammatory cascades encompassing TNF-α and interleukins, adipokines, and leptin, potentially predisposing to hepatotoxicity (40). Parallelly, findings from animal experiments have suggested that hypertension-induced liver injury may emanate from the confluence of anomalies in hepatic fatty acid metabolism and concurrent microcirculatory disturbances (41). These mechanistic determinants, among others, beckon additional rigorous scrutiny to validate their veracity.

The present study conducted a comprehensive investigation with a large sample size to explore the association between the sarcopenia index and abnormal liver function, while rigorously adjusting for potential confounding factors to ensure the reliability of the results. However, the study does have some limitations. Firstly, due to its cross-sectional design, it is not possible to establish a causal relationship, and further prospective cohort studies are needed to confirm causality. Secondly, there is currently no universally accepted definition for the sarcopenia index. Thirdly, there may still be unaccounted confounding factors that could potentially bias the accurate assessment of the true associations.

## 5 Conclusion

This cross-sectional study yields robust evidence indicating a negative correlation between the sarcopenia index and abnormal liver function, predominantly observed among females. Consequently, individuals with lower sarcopenia index values should consider enhancing their physical exercise regimens, increasing protein intake, and managing underlying health conditions such as diabetes, inflammatory bowel disease, and renal disorders, which may contribute to muscle loss. These measures can lead to an improvement in sarcopenia index and potentially help mitigate the risk of abnormal liver function.

## Data availability statement

The datasets presented in this study can be found in online repositories. The names of the repository/repositories and accession number(s) can be found in the article/supplementary material.

## Ethics statement

The studies involving humans were approved by the National Center for Health Statistics. The studies were conducted in accordance with the local legislation and institutional requirements. The participants provided their written informed consent to participate in this study. Written informed consent was obtained from the individual(s) for the publication of any potentially identifiable images or data included in this article.

## Author contributions

JX: Conceptualization, Data curation, Formal analysis, Investigation, Methodology, Project administration, Validation, Writing – original draft. Z-XX: Data curation, Software, Writing – review & editing. Q-FY: Resources, Software, Writing – review & editing. JZ: Data curation, Resources, Writing – review & editing. XZ: Resources, Visualization, Writing – review & editing. JY: Conceptualization, Supervision, Writing – review & editing.

## Footnotes

^1^ https://www.cdc.gov/nchs/nhanes/index.htm
